# Association between a body shape index and prostate cancer: a cross-sectional study of NHANES 2001–2018

**DOI:** 10.1007/s11255-023-03917-2

**Published:** 2024-01-12

**Authors:** Xiaowu Liu, Honglei Shi, Yunfeng Shi, Hanping Wei, Xiaoliang Yuan, Zhimin Jiao, Tingchun Wu, Zengjun Wang

**Affiliations:** 1 Department of Urology, First Affiliated Hospital of Nanjing Medical University, Nanjing, 210029 Jiangsu China; 2 Department of Urology, Wujin Hospital Affiliated with Jiangsu University, The Wujin Clinical College of Xuzhou Medical University, Changzhou, 213000 Jiangsu China

**Keywords:** A body shape index, Obesity, Visceral fat, Prostate cancer

## Abstract

**Objective:**

Abdominal obesity, especially visceral fat, may have negative effects on the development and progression of prostate cancer (PCa). A body shape index (ABSI) can more accurately measure visceral fat accumulation. This study aimed to investigate the association between ABSI and PCa in US adults.

**Methods:**

11,013 participants were enrolled in the National Health and Nutrition Examination Survey from 2001 to 2018. Weighted multivariate logistic regression analyses were employed to explore the independent relationship between ABSI and PCa. Moreover, restricted cubic spline (RCS) analysis, subgroup analysis, and interaction tests were performed.

**Results:**

ABSI was positively associated with the presence of PCa. When comparing the second, third, and fourth ABSI quartile to the lowest quartile, the adjusted odds ratios (95% confidence intervals) for PCa risk were 1.34 (0.77, 2.31), 1.75 (1.03, 3.00), and 1.91 (1.12, 3.27), respectively (*p* for trend = 0.011). The restricted cubic spline regression analysis did not reveal a non-linear correlation between ABSI and PCa (*p* for non-linearity = 0.076). Subgroup analysis showed a significant interaction effect in subgroups of different BMI (*p* for interaction = 0.01).

**Conclusions:**

Elevated ABSI is significantly associated with an increased risk of PCa, particularly among individuals who are under/normal weighted or obese.

**Supplementary Information:**

The online version contains supplementary material available at 10.1007/s11255-023-03917-2.

## Introduction

Globally, prostate cancer (PCa) is the second most frequently diagnosed cancer among males and ranks fifth in terms of cancer-related mortality rates [1]. According to the recent data from the American Cancer Society, there would be 268,490 new PCa cases diagnosed, and 33,500 deaths resulting from it in the United States in 2022 [2]. Unfortunately, in spite of the high incidence and morbidity of PCa, its etiology is not yet clearly elucidated. The occurrence and development of PCa is a complex, multistage process, and the environment, diet, lifestyle, and genetics are all involved in the risk of PCa [3, 4].

Obesity has been proven to be associated with 13 cancer types, which tends to replace tobacco as the primary preventable risk factor [5, 6]. For the PCa population, strong evidence indicates that obesity leads to an increase in the risk of relapse after curative-intent treatment, cancer progression, and cancer-specific death [7]. Nonetheless, the relationship between obesity with PCa incidence remains complex and inadequately defined. Additionally, most of the existing reports elucidating the relationship apply body mass index (BMI) as the standard clinical indicator for obesity [8, 9]. While BMI has been the regular method for decades to evaluate obesity, it fails to reflect fat distribution and visceral fat, which may play a more important role in the development and progression of PCa and better explain the different confirmed associations between obese patients with their clinical outcome [10, 11].

A body shape index (ABSI) is a novel anthropometric index for measuring abdominal obesity and visceral fat, first developed in 2012 [12]. Statistically, ABSI is independent of height, BMI, and waist circumference (WC) and can better reflect the degree of visceral obesity than traditional anthropometric indexes. The available evidence has shown the utility of ABSI for the prediction of cancer risk, which is restricted to a few cancer types, such as colorectal and breast cancer [13, 14]; however, limited studies have evaluated the association between ABSI with PCa risk until now.

Thus, the current study was designed to characterize the correlation between ABSI and the incidence of PCa on the basis of individuals’ data from the National Health and Nutrition Examination Survey (NHANES) database.

## Methods

### Study design and population

NHANES is a continuous U.S.-representative cross-sectional survey utilizing a stratified, multistage probability sampling design, which is conducted by the National Center for Health Statistics. The survey protocol was reviewed and approved by the National Center for Health Statistics Research Ethics Review Board, and all survey participants signed written informed consent documents [15]. A more detailed description of the design methodology and data collection can be reached on the NHANES website (http://www.cdc.gov/nchs/nhanes.htm). In this study, we integrated data for analysis from nine two-year NHANES cycles (2001–2018).

**Fig. 1:**
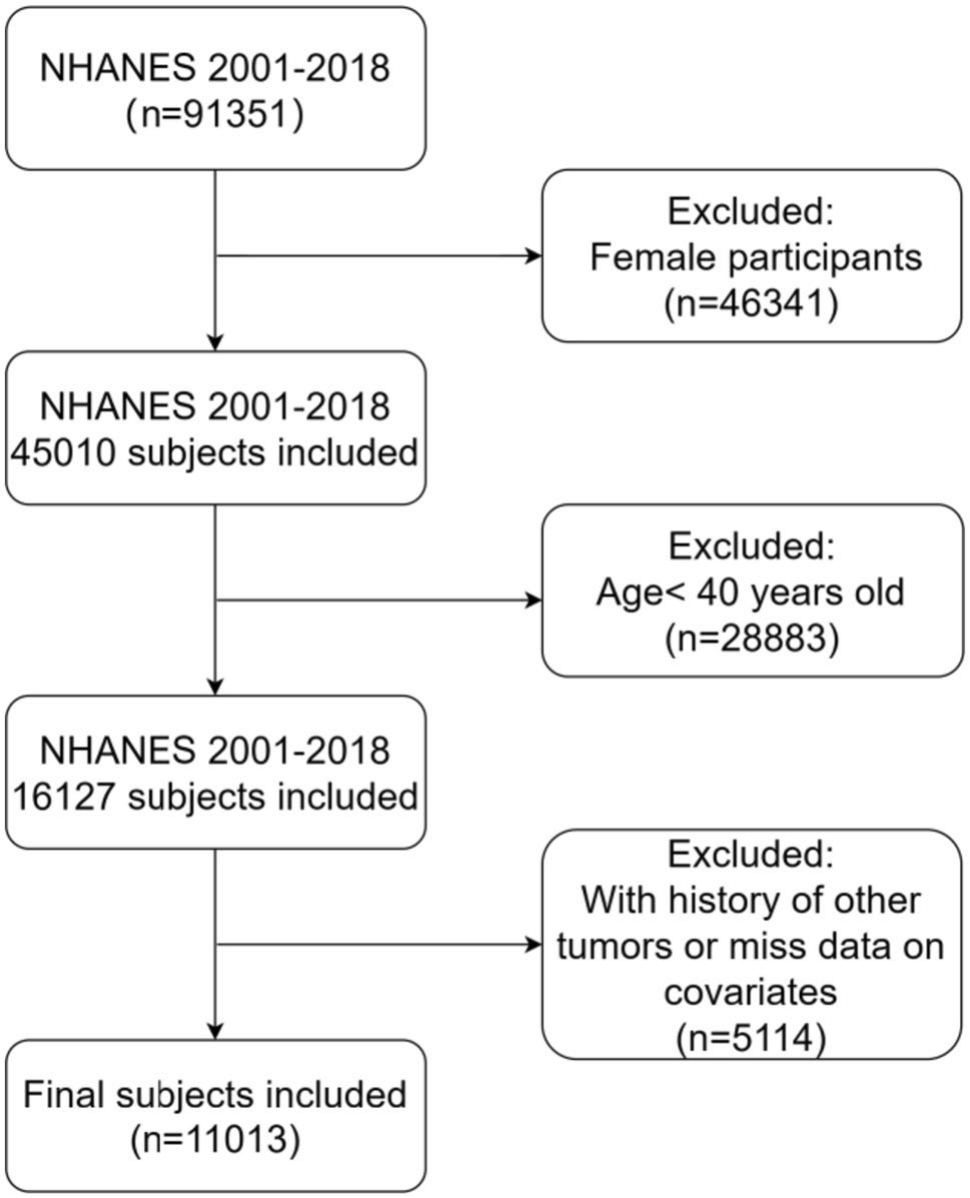
The flowchart in selecting the studying participants

The inclusion criteria for all individuals were men aged ≥ 40 years old, with no other tumor history except PCa. Ultimately, after excluding participants with missing data for important covariates, a total of 11,013 participants were enrolled (Fig. 1).

### Variables

The dependent variable was PCa, and the independent variable in this research was ABSI. The questionnaire items, “Ever been told you had cancer or a malignancy of any kind” and “What kind of cancer was it” were used to identify individuals with PCa. ABSI was calculated by $1000\times{\text{WC}}\;{\text{(m)}} \times {\text{height}}\;{\text{(m)}}^\frac{5}{6} \times {\text{weight}}\;{\text{(kg}})^{ - \frac{2}{3}}$ [12, 16]. Demographic characteristics on age, gender, race, education level (less than high school and high school or above), family income level, and marital status were evaluated. Age was divided into three groups: < 60, 60–80, and ≥ 80 years. Based on the poverty income ratio (PIR), family income level was classified into three levels: low (PIR ≤ 1), middle (1 < PIR < 3), and high (PIR ≥ 3). According to marital status options, living status was categorized into living alone (“widowed,” “divorced,” “separated,” or “never married”), and living with a partner(“married” or “living with a partner”) [17]. Lifestyle factors and health conditions included smoking status, drinking status, BMI, Hypertension, and diabetes (DM). Smoking status was classified as never smoker who had never smoked or smoked < 100 cigarettes in life, former smoker who had smoked ≥ 100 cigarettes in life and smoked not at all currently, and current smoker who had smoked ≥ 100 cigarettes in life and was still smoking regularly [18]. Drinking status was classified as heavy drinker (≥ 3 drinks per day, or binge drinking ≥ 5 drinks on 5 or more days per month), never drinker (< 12 drinks in lifetime), and mild drinker [19, 20]. BMI was categorized as under/normal weight (BMI < 25), overweight (25 ≤ BMI < 30), and obese BMI ≥ 30). Participants with DM were identified by one of the following criteria: self-reported diabetes, hemoglobin A1c (HbA1c) ≥ 6.5%, fasting glucose ≥ 7.0 mmol/l, 2-h oral glucose tolerance test/random blood glucose ≥ 11.1 mmol/l, and use of antidiabetic medication. Participants who met one of the following criteria were defined as hypertension patients, including self-reported hypertension, use of antihypertensive drugs, systolic blood pressure ≥ 140 mmHg, and diastolic blood pressure ≥ 80 mmHg [21].

### Statistical analysis

Statistical analysis was conducted by taking sample weights into consideration, according to the Centers for Disease Control and Prevention (CDC) guidelines. Univariate analysis was first performed to evaluate baseline characteristics among participants. Categorical variables are expressed as percentages (%) and analyzed by weighted chi-squared test, while continuous variables are presented as mean ± standard deviation (SD) and analyzed by weighted *t*-test. Secondly, weighted multivariate logistic regression was utilized to investigate the association between ABSI and PCa, and three statistical models were constructed: model I, adjusted with no covariates; model II, just adjusted for demographic covariates only; and model III, adjusted for all covariates. The same approach was employed to explore the relationship in the ABSI quartiles, with the lowest quartile as the reference. The final results are represented as odds ratios (OR) and 95% confidence intervals (CIs). Further, to explore the potential non-linear association between ABSI and PCa, restricted cubic spline (RCS) analyses were performed with knots at the 5th, 50th, and 95th percentiles [22]. Lastly, subgroup analyses stratified by different confounders were also conducted.

R 4.3.1 (https://www.r-project.org/) was used to carry out all of the data analysis. A two-sided *p* < 0.05 was considered statistically significant.

## Results

### Baseline characteristics of study participants

Baseline characteristics of participants selected from NHANES 2001 to 2018 are shown in Table 1. A total of 11,013 participants were enrolled in this study, 492 with PCa and 10,521 without PCa. Between two groups with or without PCa, ABSI, WC, height, age, race, family income level, drinking status, smoking status, hypertension, and diabetes were all significantly different (all *p* < 0.05), while other characteristics including weight, education level, living status, and BMI had no statistically significant differences.

**Table 1:** Baseline characteristics of the selected participants in NHANES 2001–2018

Characteristics	Total (*n* = 11,013)	Non PCa (*n* = 10,521)	PCa (*n* = 492)	*p* value
ABSI	83.40 ± 0.06	83.32 ± 0.06	85.93 ± 0.22	< 0.001
WC (cm)	104.36 ± 0.22	104.29 ± 0.23	106.48 ± 0.94	0.026
Height (cm)	175.65 ± 0.12	175.71 ± 0.13	173.80 ± 0.49	< 0.001
Weight (cm)	90.23 ± 0.27	90.30 ± 0.27	87.91 ± 1.36	0.091
Age group, *n* (%)^a^				
< 60	5790 (67.27)	5758 (69.06)	32 (10.24)	< 0.001
60–80	4425 (29.03)	4086 (27.76)	339 (69.39)	
≥ 80	798 (3.70)	677 (3.18)	121 (20.36)	
Race, *n* (%)^a^				< 0.001
Non-Hispanic white	5050 (73.19)	4783 (73.09)	267 (76.41)	
Non-Hispanic black	2398 (9.84)	2243 ( 9.67)	155 (15.11)	
Mexican American	1798 (6.66)	1774 (6.81)	24 (1.87)	
Other race	925 (6.01)	907 (6.08)	18 (3.72)	
Other Hispanic	842 (4.30)	814 (4.35)	28 (2.88)	
Education level, *n* (%)^a^				0.075
≤ High school level	5690 (41.28)	5459 (41.46)	231 (35.80)	
> High school level	5323 (58.72)	5062 (58.54)	261 (64.20)	
Family income level, *n* (%)^a^				
Low	1898 (10.09)	1842 (10.20)	56 (6.68)	0.046
Middle	4525 (32.00)	4293 (31.83)	232 (37.42)	
High	4590 (57.91)	4386 (57.97)	204 (55.89)	
Living status, *n* (%)^a^				0.777
Live alone	3080 (24.31)	2942 (24.34)	138 (23.54)	
Live with partner	7933 (75.69)	7579 (75.66)	354 (76.46)	
BMI, *n* (%)^a^				0.372
Under/normal weight	2605 (20.83)	2484 (20.79)	121 (21.95)	
Overweight	4530 (41.61)	4320 (41.51)	210 (44.62)	
Obese	3878 (37.56)	3717 (37.69)	161 (33.43)	
Drinking status, *n* (%)^a^				< 0.001
Never	801 (5.92)	754 (5.89)	47 (6.88)	
Mild	8164 (75.06)	7750 (74.65)	414 (88.06)	
Heavy	2048 (19.02)	2017 (19.46)	31 (5.06)	
Smoking status, *n* (%)^a^				< 0.001
Never	4446 (44.04)	4253 (44.20)	193 (38.97)	
Former	4054 (34.86)	3807 (34.32)	247 (52.15)	
Current	2513 (21.10)	2461 (21.48)	52(8.88)	
Hypertension, *n* (%)^a^				< 0.001
No	5104 (52.06)	4970 (52.65)	134 (33.13)	
Yes	5909 (47.94)	5551 (47.35)	358 (66.87)	
Diabetes, *n* (%)^a^				< 0.001
No	8261 (80.89)	7918 (81.27)	343 (69.01)	
Yes	2752 (19.11)	2603 (18.73)	149 (30.99)	

Data are expressed as mean ± SD for continuous variables or as percentages for categorical variables. *p* value was calculated by weighted *t*-test and weighted chi-square test

*PCa* prostate cancer, *ABSI* a body shape index, *WC* waist circumference, *BMI* body mass index

^a^Unweighted frequency counts and weighted percentages are shown

### Associations between BRI and PCa

The construction of univariate and multivariate logistic regression models is presented in Table 2. Model I was adjusted for no covariates, while Model II adjusted for age, race, education level, family income level, and living status. Model III was further adjusted for BMI, drinking status, smoking status, hypertension, and diabetes. All of the models mentioned above demonstrated a positive correlation between ABSI and PCa, with all p values less than 0.05. The odds ratios (95% confidence intervals) for model I, model II, and model III were 1.16 (1.14, 1.19), 1.05 (1.02, 1.08), and 1.05 (1.02, 1.08), respectively. To further conduct a sensitivity analysis and assess the trend, ABSI was categorized into quartiles in Table 2. In model III, when comparing the Q2, Q3, and Q4 groups to the reference Q1 group, the adjusted odds ratios (95% confidence intervals) for PCa risk were 1.34 (0.77, 2.31), 1.75 (1.03, 3.00), and 1.91 (1.12, 3.27), respectively (*p* for trend = 0.011). Similarly, these trends persisted in model I (*p* for trend < 0.001) and model II (*p* for trend = 0.005).

**Table 2:** Association between ABSI and PCa in NHANES 2001–2018

	Model I^a^		Model II^b^		Model III^c^	
	OR (95% CI)	*p* value	OR (95% CI)	*p* value	OR (95% CI)	*p* value
ABSI	1.16 (1.14, 1.19)	< 0.001	1.05 (1.02, 1.08)	0.001	1.05 (1.02, 1.08)	0.003
ABSI quartiles						
Q1 (66.74, 80.94)	Ref		Ref		Ref	
Q2 (80.94, 83.66)	1.92 (1.14, 3.26)	0.015	1.32 (0.77, 2.27)	0.313	1.34 (0.77, 2.31)	0.294
Q3 (83.66, 86.42)	3.69 (2.24, 6.06)	< 0.001	1.76 (1.04, 2.97)	0.035	1.75 (1.03, 3.00)	0.040
Q4 (86.42, 116.59)	6.43 (3.96, 10.42)	< 0.001	1.92 (1.15, 3.23)	0.014	1.91 (1.12, 3.27)	0.019
*p* for trend	< 0.001		0.005		0.011	

*ABSI* a body shape index

^a^Model I was adjusted for no covariates

^b^Model II was adjusted for age, race, education level, family income level, and living status

^c^Model III was adjusted for age, race, education level, family income level, living status, BMI, drinking status, smoking status, hypertension, and diabetes

### Identification of non-linear relationship

**Fig. 2:**
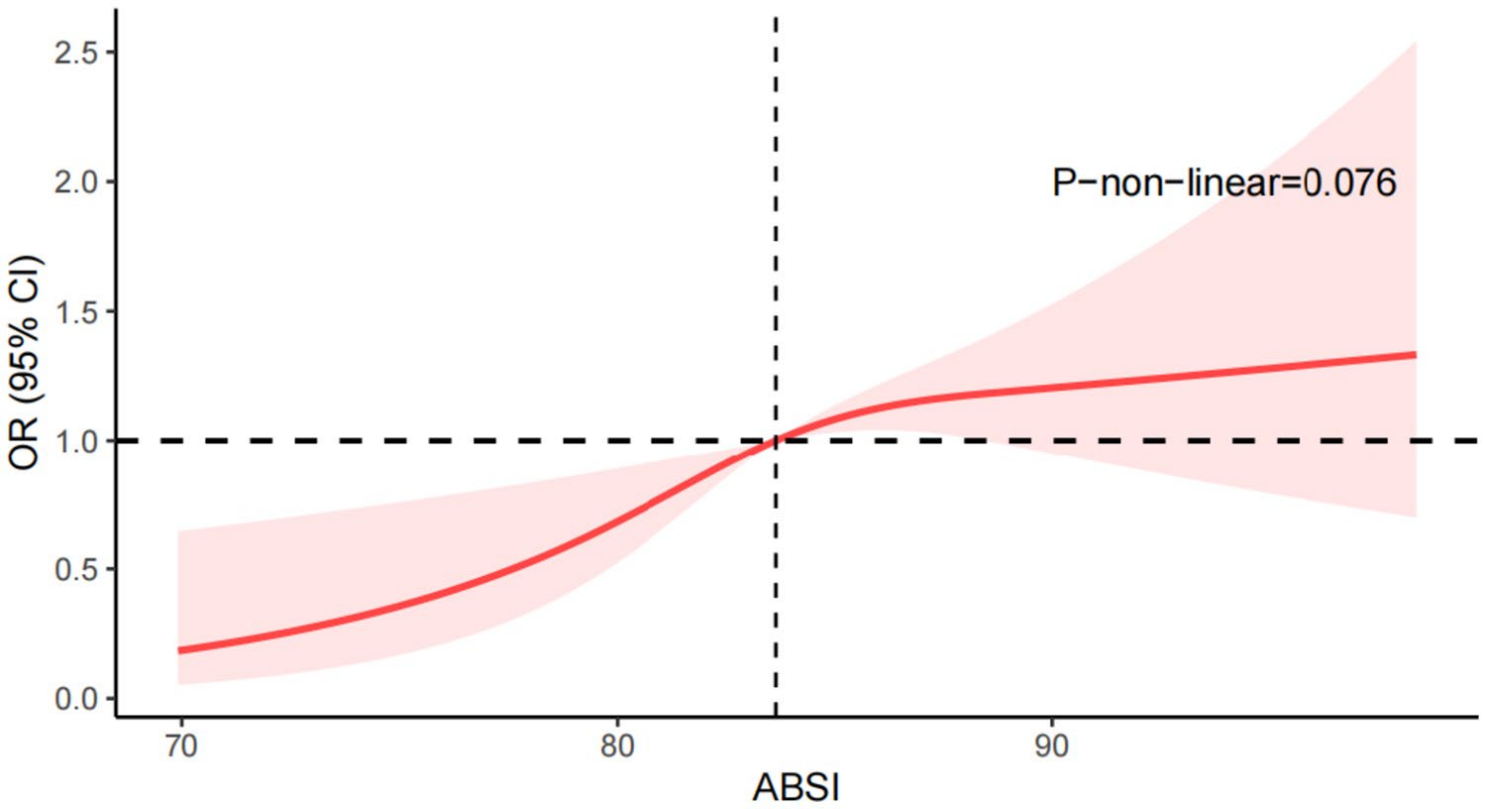
Relationship between ABSI and the odds ratio of PCa. The restricted cubic spline regression model was adjusted for age, race, education level, family income level, living status, BMI, drinking status, smoking status, hypertension, and diabetes.

To identify and visualize the non-linear relationship between ABSI and PCa, data were fitted by a restricted cubic spline regression model (Fig. 2). The result showed that there was no non-linear correlation between ABSI and PCa (*p* for non-linearity = 0.076), and the adjusted ORs for PCa rose along with increasing ABSI.

### Subgroup analysis

**Fig. 3:**
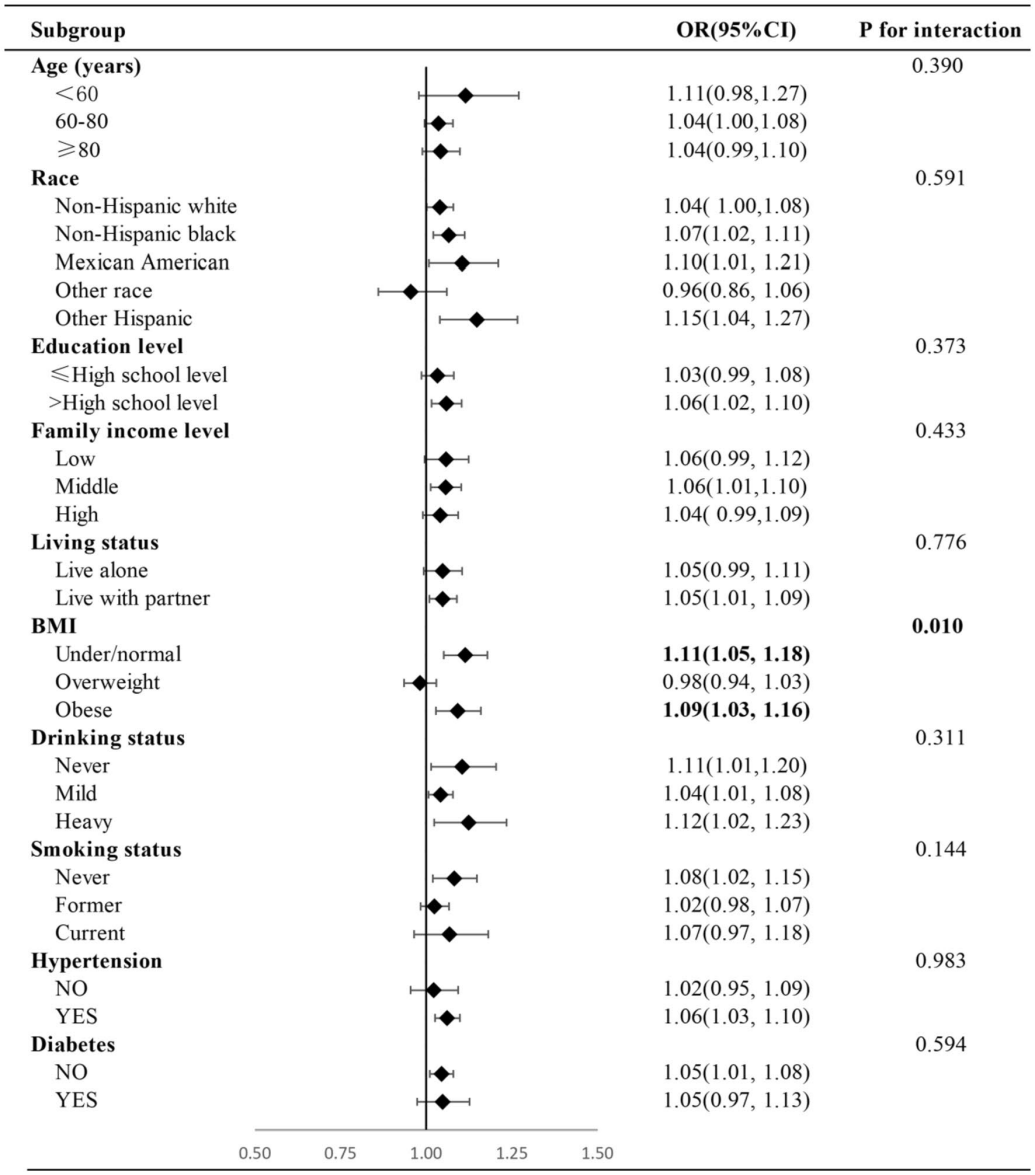
Subgroup analysis of the association between ABSI and PCa. All subgroup analysis was adjusted for age, race, education level, family income level, living status, BMI, drinking status, smoking status, hypertension, and diabetes, except for the covariate defining the subgroup.

The results of subgroup analysis stratified by age, race, education level, family income level, living status, BMI, drinking status, smoking status, hypertension, and diabetes are shown in Fig. 3. All associations were positive in the different subgroups, except for subsets of other race and with overweight. Of note, for the BMI subgroup, there was a significant interaction with the association between ABSI and PCa (*p* for interaction = 0.01), while not among other subgroups (*p* for interaction > 0.05). Additionally, stronger positive associations were found in individuals with under/normal weight (OR (95% CI), 1.11 (1.05, 1.18)), while the associations were weakened in obese individuals (OR (95% CI), 1.09 (1.03, 1.16)).

## Discussion

In this cross-sectional study that enrolled 11,013 participants from NHANES 2001–2018, we observed a positive association between the ABSI and PCa, appearing to be a linear dose–response relationship. Moreover, in the BMI subgroup analyses, there was a significant interaction between the association; individuals of under/normal weight with a higher ABSI showed a higher risk of PCa than obese ones. The present results suggested that visceral fat control might be conducive to preventing PCa.

Visceral obesity has been found to possess a greater pro-oncogenic potential compared to total body fat, and it is also associated with various cardiometabolic conditions including insulin resistance, diabetes, hypertension, and metabolic syndrome [23]. Chronic low-level inflammation induced by excess fat mass can cause immune imbalance and metabolic imbalance resulting from the deregulation of adipokines signaling, abnormal concentrations of sex hormone, and alterations in insulin and insulin-like growth factor (IGF) axis; consequently, this state may promote a favorable tumor micro-environment favorable for cancer cell growth [24, 25]. The precise mechanisms underlying the correlation between the accumulation of visceral obesity and PCa remain incompletely elucidated. The surplus adipose tissue can activate inflammatory cytokines, such as tumor necrosis factor-alpha (TNF-α) and interleukin 6 (IL-6), as well as secrete leptin, all of which have been linked to PCa [24, 26]. Furthermore, the accumulation of visceral obesity leads to insulin resistance, resulting in elevated levels of insulin and IGF-1, both of which further contribute to the risk of developing prostate cancer [7, 24]. A prospective study of 1832 men demonstrated that greater visceral fat measured through computed tomography (CT) was associated with prostate cancer aggressiveness [10]. Similarly, Dickerman et al. [11] examined peri-prostatic fat volume (PPFV) by magnetic resonance imaging (MRI) and showed those with greater peri-prostatic fat had a risk for developing castrate-resistant prostate cancer faster. Interestingly, in both studies, there were no associations between total body fat mass with any prostate cancer-related outcomes, indicating that fat type and distribution may be of great significance [10, 11].

Overall obesity was often defined using BMI [27], and there is even “normal weight obesity” in the population. Even for under- or normal-weight individuals, the risk for PCa may be higher among those with greater visceral fat, just as with the subgroup analysis. Furthermore, previous studies widely utilized the waist-to-height ratio or WC to evaluate visceral obesity, which was unable to differentiate between visceral fat and subcutaneous fat [23, 28–30]. Hence, ABSI, a new anthropometric indicator, was introduced to measure visceral fat; meanwhile, it is more feasible and cheaper in routine clinical practice and overall population than CT or MRI.

ABSI has been found to be a reliable indicator of visceral fat accumulation [31], making it a suitable tool for identifying populations with cardiovascular disease risk factors and predicting cardiovascular mortality [32]. Also, ABSI was demonstrated as an independent predictor for metabolic syndrome among both obese children and adolescents [33]. Moreover, an increasing body of research has endeavored to examine the relationship between ABSI and cancer risks. Notably, a study conducted on the UK Biobank cohort demonstrated significant positive associations between ABSI and the risk of developing liver, lung, colorectal cancer, as well as all cancer types combined [13, 34]. Furthermore, a separate investigation involving 143,901 women in the United States revealed that ABSI was not associated with breast or endometrial cancer risk. Conversely, concerning urologic neoplasms, a higher ABSI was found to be linked to an elevated risk of bladder cancer in men [13] and kidney cancer in women [13, 35]. Nevertheless, existing evidence for the association between ABSI and PCa remains sparse. Based on a cohort study of Swedish residents, ABSI was not associated with risk for PCa or PCa-specific death [16]. Similarly, in the pooled collaborative analysis of 11 Australian cohorts [36], encompassing 79,458 participants, no statistically significant association was found between ABSI and prostate cancer incidence, which was inconsistent with our result. Our findings showed a higher risk of PCa in the underweight/normal population with elevated ABSI, which suggested the need to further focus on visceral fat control rather than body weight alone. The inconsistency could be due to several factors, including differences in population, sample sizes, study designs, and statistical methods. Thus, further studies are required to evaluate the diagnostic value of ABSI for PCa and assess whether ABSI can be applied in PCa screening considering overdiagnosis.

The present study represented the initial investigation into the correlation between ABSI and PCa within a substantial and representative national cohort of American adults. Our study possessed several strengths, including meticulous study protocols and quality controls, a sizable and representative sample, and comprehensive data on numerous vital covariates through the integration of NHANES data. However, it is important to acknowledge certain limitations of this study. Firstly, the NHANES did not collect imaging and pathological data from its participants, and thus, we relied on self-reported physician-diagnosed cases of PCa to define individuals with PCa. Some recall bias should be considered. Secondly, our study was a cross-sectional analysis, which inherently limited the ability to establish a definitive causal relationship. Additionally, the lack of follow-up data for ABSI and PCa patients from NHANES further contributed to the uncertainty surrounding the temporal relationship between alterations in ABSI and the prognosis of PCa. Thirdly, due to the limitations in the NHANES database, we were unable to obtain clinical data on the grade, stage, and treatment of PCa among the participants, thereby impeding the stratified evaluation of potential variations in the association between ABSI and the risk or progression of PCa. Fourthly, the potential impact of modifications in prostate cancer screening strategies, which may lead to variations in PCa incidence during that particular stage, has been overlooked.

## Conclusions

This study suggests a significant association between elevated ABSI and an increased risk of PCa, particularly among individuals who are under/normal weighted or obese. ABSI holds promise as a feasible tool for clinical evaluation of PCa risk; however, a cross-sectional study cannot establish causal effects, and further exploration is needed. The objective of this study is to enhance public understanding of the relationship between visceral fat indicated by ABSI and cancer, as well as to promote effective measures such as dietary regulation, regular physical activity, and other interventions to reduce body weight, specifically targeting visceral fat accumulation.

### Supplementary Information

Below is the link to the electronic supplementary material.

Supplementary file1 (DOCX 17 kb)

Supplementary file2 (DOCX 12 kb)

Supplementary file3 (DOCX 122 kb)
