# Supplementary material for: Association between a body shape index and prostate cancer: a cross-sectional study of NHANES 2001–2018
Source: Int Urol Nephrol. 2024 Jan 12;56(6):1869–77. doi: 10.1007/s11255-023-03917-2 (PMC11090932; doi:10.1007/s11255-023-03917-2)
Supplement: Supplementary file 1 — Supplementary file1 (DOCX 17 kb) [file 11255_2023_3917_MOESM1_ESM.docx]

**Supplementary Table 1** Association between traditional anthropometric measures and PCa in NHANES 2001–2018.

|  | Model Ⅰ^a^ | | Model Ⅱ^b^ | | Model Ⅲ^c^ | |
| --- | --- | --- | --- | --- | --- | --- |
|  | OR(95%CI) | P value | OR(95%CI) | P value | OR(95%CI) | P value |
| WC | 1.01(1.00,1.02) | **0.020** | 1.01(1.00, 1.02) | 0.261 | 1.00(0.99, 1.01) | 0.700 |
| BMI(Continuous) |  |  |  |  |  |  |
| BMI(Categories) | 0.99(0.97,1.02) | 0.674 | 1.01(0.98, 1.04) | 0.520 | 1.00(0.97, 1.03) | 0.896 |
| Under/normal | ref |  | ref |  | ref |  |
| Overweight | 1.02(0.78,1.33) | 0.893 | 1.11( 0.83, 1.48) | 0.490 | 1.03( 0.76, 1.39) | 0.856 |
| Obese | 0.84(0.59,1.19) | 0.323 | 0.98( 0.67, 1.44) | 0.931 | 0.84( 0.54, 1.30) | 0.430 |
| P for trend | 0.269 | | 0.843 | | 0.376 | |

Abbreviation: WC, waist circumference; BMI, [body mass index](https://www.sciencedirect.com/topics/medicine-and-dentistry/body-mass-index).

^a^Model Ⅰ was adjusted for no covariates.

^b^Model Ⅱ was adjusted for age, race, education level, family income level, and living status.

^c^Model Ⅲ was adjusted for age, race, education level, family income level, living status, drinking status, smoking status, hypertension, and diabetes.
