# Supplementary material for: Association between a body shape index and prostate cancer: a cross-sectional study of NHANES 2001–2018
Source: Int Urol Nephrol. 2024 Jan 12;56(6):1869–77. doi: 10.1007/s11255-023-03917-2 (PMC11090932; doi:10.1007/s11255-023-03917-2)
Supplement: Supplementary file 2 — Supplementary file2 (DOCX 12 kb) [file 11255_2023_3917_MOESM2_ESM.docx]

**Supplementary Table 2** Association between ABSI and PCa after excluding participants from NHANES cycles.

|  | Model Ⅰ^a^ | | Model Ⅱ^b^ | | Model Ⅲ^c^ | |
| --- | --- | --- | --- | --- | --- | --- |
|  | OR(95%CI) | P value | OR(95%CI) | P value | OR(95%CI) | P value |
| Excluding 2001-2002 | 1.16(1.13,1.19) | **<0.001** | 1.05(1.02, 1.08) | **0.003** | 1.04(1.01, 1.08) | **0.007** |
| Excluding 2003-2004 | 1.16(1.13,1.19) | **<0.001** | 1.05(1.02, 1.08) | **0.002** | 1.05(1.01, 1.08) | **0.007** |
| Excluding 2005-2006 | 1.17(1.14,1.20) | **<0.001** | 1.05(1.02, 1.08) | **0.002** | 1.05(1.01, 1.08) | **0.005** |
| Excluding 2007-2008 | 1.16(1.13,1.19) | **<0.001** | 1.04(1.01, 1.08) | **0.008** | 1.04(1.01, 1.08) | **0.016** |
| Excluding 2009-2010 | 1.16(1.13,1.19) | **<0.001** | 1.05(1.01, 1.08) | **0.005** | 1.04(1.01, 1.08) | **0.012** |
| Excluding 2011-2012 | 1.17(1.14,1.20) | **<0.001** | 1.06(1.03, 1.09) | **<0.001** | 1.05(1.02, 1.08) | **<0.001** |
| Excluding 2013-2014 | 1.17(1.14,1.21) | **<0.001** | 1.05(1.01, 1.08) | **0.005** | 1.05(1.01, 1.08) | **0.007** |
| Excluding 2015-2016 | 1.16(1.13,1.19) | **<0.001** | 1.04(1.01, 1.07) | **0.019** | 1.04(1.00, 1.07) | **0.027** |
| Excluding 2017-2018 | 1.17(1.14,1.21) | **<0.001** | 1.07(1.04, 1.10) | **<0.001** | 1.07(1.04, 1.10) | **<0.001** |

^a^Model Ⅰ was adjusted for no covariates.

^b^Model Ⅱ was adjusted for age, race, education level, family income level, and living status.

^c^Model Ⅲ was adjusted for age, race, education level, family income level, living status, drinking status, smoking status, hypertension, and diabetes.
