# Supplementary material for: Association between a body shape index and prostate cancer: a cross-sectional study of NHANES 2001–2018
Source: Int Urol Nephrol. 2024 Jan 12;56(6):1869–77. doi: 10.1007/s11255-023-03917-2 (PMC11090932; doi:10.1007/s11255-023-03917-2)
Supplement: Supplementary file 3 — Supplementary file3 (DOCX 122 kb) [file 11255_2023_3917_MOESM3_ESM.docx]

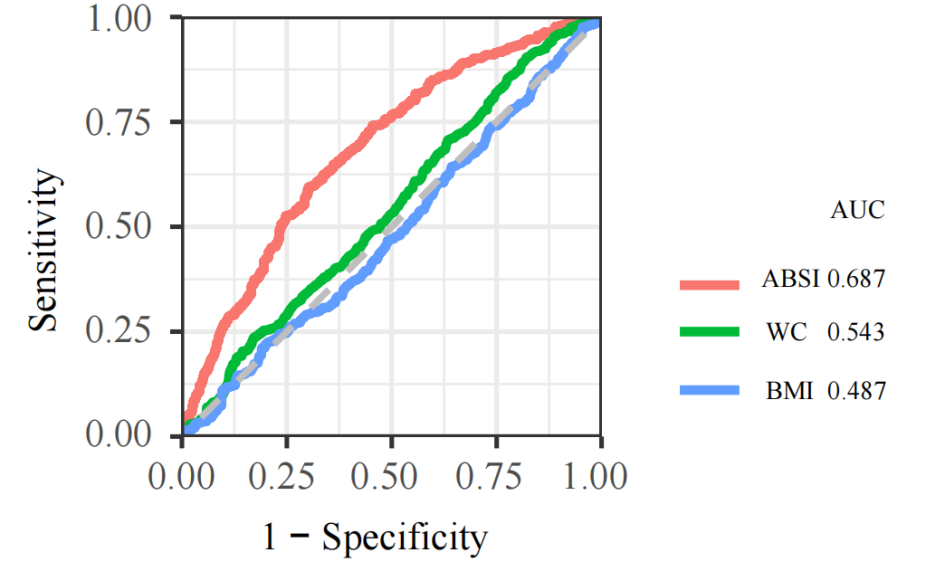


**Supplementary Figure 1** ROC curve for ABSI、WC、BMI to predict PCa.

Abbreviation: WC, waist circumference; BMI, body mass index; ROC curve, receiver operating characteristic curve;AUC, area under the ROC curve.
